# Bouveret Syndrome in a Cirrhotic Patient

**DOI:** 10.7759/cureus.32270

**Published:** 2022-12-06

**Authors:** Daniel T Gildea, Dalal Alhaqqan, Krystina Johnson, Amol S Rangnekar

**Affiliations:** 1 Internal Medicine, MedStar Georgetown University Hospital, Washington, D.C., USA; 2 Gastroenterology and Hepatology, MedStar Georgetown University Hospital, Washington, D.C., USA

**Keywords:** bouveret syndrome, gallstone ileus, gallbladder, esophagogastroduodenoscopy (egd), cholethiasis, gallstone, endoscopy

## Abstract

Here, we present a case of Bouveret syndrome, a rare etiology of gallstone impaction in the setting of chole-enteric fistula, in a cirrhotic patient. This syndrome is most often seen in elderly patients with multiple comorbidities and as such has high morbidity and mortality rates. Because of its prevalence in this patient population and its rarity, there are no established guidelines for the workup and management of this disease. We discuss currently available options for management and thoughts on our comorbid patient and her clinical course.

## Introduction

Bouveret syndrome is characterized by a gastric outlet or duodenal obstruction secondary to stone impaction in the pylorus or duodenum in the setting of cholecystogastric, cholecystocolic, or most commonly cholecystoduodenal fistula. It is a very rare complication of cholelithiasis seen in 0.3%-0.5% of gallstone cases [1]. Despite its rarity, risk factors have been studied and identified; they include a history of cholelithiasis, stones greater than 2-8 cm, female gender, and age > 60 years. Due to its disproportionate presentation in the elderly, complexity, and nonspecific presentation in combination with no set guidelines in terms of workup and management, it has high morbidity and mortality rates, with mortality estimates ranging from 12% to 30% [1]. Bouveret syndrome often presents after a bout of cholecystitis (more rarely gallbladder malignancy), with fistula development thought to be occurring secondary to gallbladder inflammation and adhesion to the gastrointestinal (GI) tract in combination with gallstone-related pressure on the gallbladder wall leading to ischemia and perforation [2]. The development of symptoms may depend on factors including gastrointestinal anatomical distortion and the size of the stone [1]. In this report, we present a case of Bouveret syndrome in an elderly female, whose management was complicated by underlying cirrhosis.

## Case presentation

**Figure 1:**
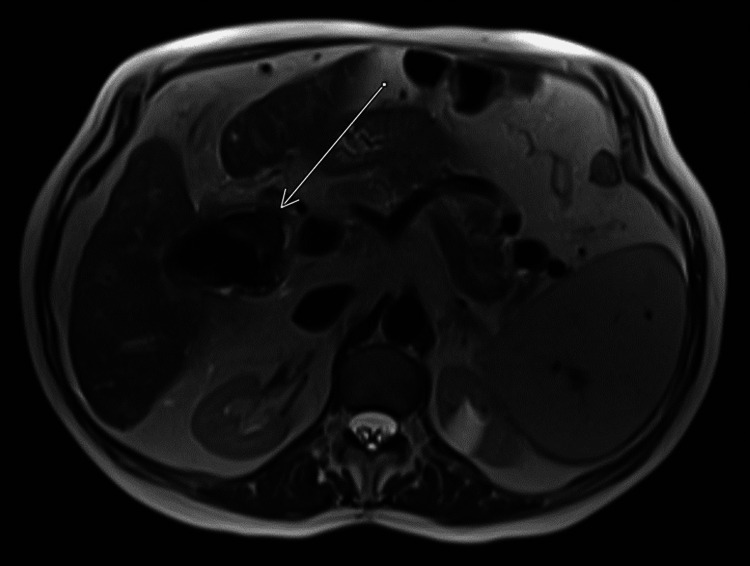
MRI demonstrating indistinct wall between the gallbladder neck and the common bile duct concerning for pneumobilia (arrow) MRI: magnetic resonance imaging

**Figure 2:**
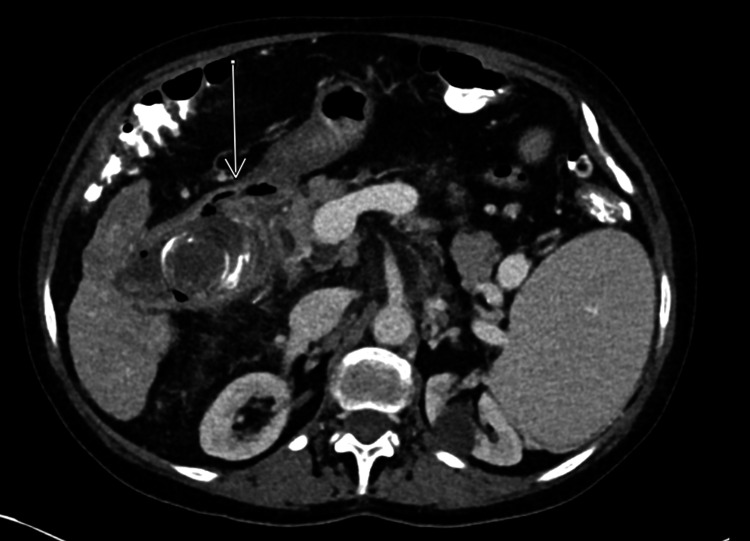
CT demonstrating fistulization of the gallbladder neck anteriorly into the duodenum (arrow) CT: computed tomography

A 75-year-old female with a history of chronic kidney disease (CKD) and decompensated cryptogenic cirrhosis presented for routine follow-up with transplant hepatology. She had no acute complaints, and a screening outpatient MRI for hepatocellular carcinoma (HCC) was ordered. Following the completion of the MRI, critical findings (Figure 1) were identified and communicated with the ordering physician: inflammatory changes of the gallbladder concerning for cholecystitis, pneumobilia, an indistinct wall between the gallbladder neck and the common bile duct, and presumed gas in the gastrointestinal lumen suspicious for a fistula to the descending duodenum, which was seen immediately adjacent to inflammatory changes of the gallbladder. The patient was contacted and reported a “gallbladder attack” after eating a meal one week prior to the MRI, consisting of sharp epigastric/right upper quadrant pain associated with nausea and one episode of emesis. She additionally reported milder postprandial “attacks” on multiple nights throughout that week. The patient was advised to present to the emergency department (ED), where she had blood work showing slightly elevated bilirubin, aspartate aminotransferase (AST), and alkaline phosphatase, as well as a CT of the abdomen/pelvis (Figure 2), disclosing a fistula between the gallbladder and the duodenum, along with stranding, gallstones, and pneumobilia, as demonstrated on MRI.

**Figure 3:**
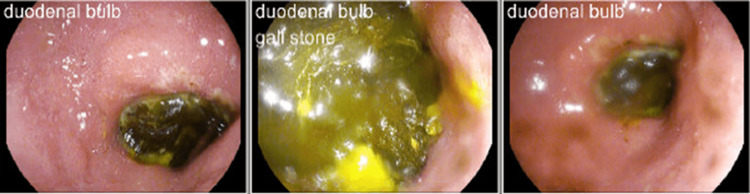
EGD demonstrating the gallstone in the duodenal bulb EGD: esophagogastroduodenoscopy

Her physical examination was unremarkable, and her vital signs were within normal limits. She was started empirically on piperacillin-tazobactam due to concern for acute cholecystitis given the recent imaging findings. After discussion with transplant surgery, the decision was made to perform a diagnostic esophagogastroduodenoscopy (EGD) for further evaluation, which demonstrated a large non-obstructive gallstone in the duodenal bulb (Figure 3), as well as esophageal varices. No therapeutic intervention was performed during EGD due to resolving symptoms as well as lack of the presence of expertise in stone removal techniques in our center. Potential interventions were discussed with general surgery. Ultimately, surgical intervention was deemed not to be appropriate at that time. The patient was discharged on a 14-day course of amoxicillin-clavulanic acid and had a follow-up with transplant hepatology. She was asymptomatic from a gallbladder perspective following discharge.

**Figure 4:**
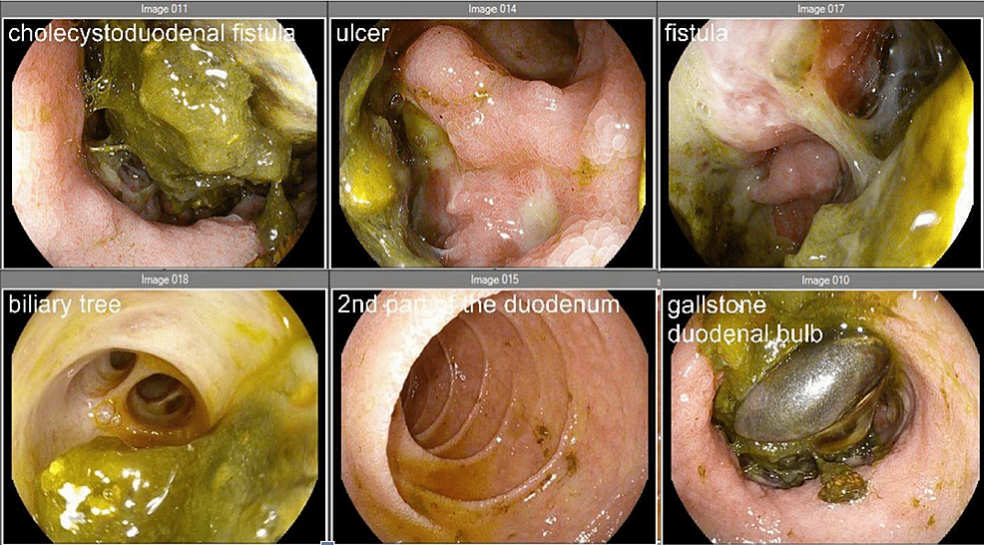
Repeat EGD demonstrating the fistula, ulcer, and gallbladder mucosa EGD: esophagogastroduodenoscopy

It was discussed during the outpatient follow-up that the patient’s case was complicated by her underlying cirrhosis; as such, she was considered a high-risk surgical candidate. Rather than immediately proceeding with cholecystectomy in the setting of such risk, a liver transplant evaluation was initiated to potentially allay this surgical risk. A transjugular intrahepatic portosystemic shunt (TIPS) procedure was strongly considered as well. Almost one year later, she presented to the ED with nausea, vomiting, and melena. Repeat EGD noted non-bleeding esophageal varices, a large partially obstructing gallstone in the duodenal bulb with ulcerated mucosa, and a persistent cholecystoduodenal fistula, which allowed visualization of the gallbladder mucosa and biliary tree (Figure 4). No intervention was performed, and the patient was discharged on proton pump inhibitor (PPI) therapy, pending multidisciplinary discussion for further management. Unfortunately, the patient passed away at home less than one month later.

## Discussion

This rare and interesting case was initially discovered incidentally via routine HCC MRI screening in a cirrhotic patient. The postprandial symptoms she describes prior to MRI, which for the most part resolved following hospitalization, likely represent intermittent obstructions relieved by the transit of the gallstone.

While there are no specific guidelines for Bouveret syndrome management, strategies for diagnosis and treatment have been developed. The initial management of Bouveret syndrome starts with making the diagnosis, which can be difficult due to its nonspecific symptoms and rarity. For instance, while our patient had preceding postprandial symptoms, it is important to note that approximately one-third of these patients have no prior biliary symptoms [3]. MRI or CT is helpful and can disclose the Rigler triad (dilated stomach, pneumobilia, and a radiopaque shadow consistent with an enteric gallstone, as well as a fistula and/or pneumobilia [4]), as seen in the patient in this case. While there is no clear “gold standard” for diagnosis, CT has been described as one of the more helpful techniques in the multimodal diagnostic approach, with a higher sensitivity than EGD [5]. EGD is often performed after the initial suspicion is present. It is used for both diagnostic and, especially given the often surgically high-risk patient population that this syndrome often presents with, therapeutic purposes.

Options for endoscopic removal include endoscopic removal with baskets or nets, mechanical lithotripsy with stone fragmentation via crushing, and even more specialized procedures including electrohydraulic lithotripsy and laser lithotripsy, both of which involve energy-focused stone fragmentation and removal [4]. Another option includes extracorporeal shockwave lithotripsy to induce fragmentation, followed by EGD to remove the gallstone pieces [4]. EGD can also be used as an adjunct to surgery if needed [4]. Surgical options are considered in the setting of a lack of technical expertise for EGD-driven removal or EGD failure. It is important for gastroenterologists to have an understanding of the risks, benefits, and likely outcomes of surgical intervention, as endoscopy has been reported as successful in less than one-third of Bouveret syndrome cases [6]. These approaches include open duodenotomy, pyloromyotomy, and gastrotomy; these open approaches have higher morbidity and mortality as compared to laparoscopic approaches such as enterolithotomy under laparoscopy [4]. It has been reported that 73% of Bouveret syndrome patients have had surgery for the disease, 61% of the time due to failed interventional endoscopy [6]. Cholecystectomy with fistula repair in these cases remains controversial. Often, as in the patient in this case, Bouveret syndrome patients are elderly with comorbidities and as such poor surgical candidates. In many of these patients, cholecystectomy is commonly not performed due to the significant risk. However, this does leave these patients at risk for recurrent gallstone episodes, as well as an increased risk of malignancy due to the unremoved fistula [4].

Other authors such as Khan et al. have created algorithms to attempt to simplify management decisions and workflow. Following initial diagnosis and stabilization, the first decision point involves patient stability. Unstable patients in this workflow are recommended to proceed to surgery, whereas stable patients undergo therapeutic EGD [7]. Patients successfully treated with EGD with fragments < 1.5 cm are simply observed, while those with fragments > 1.5 cm are considered for early surgical management. Patients with unsuccessful EGD, as well as unstable patients, as previously mentioned, would be recommended for surgery. For high-risk patients, the surgical recommendation is enterolithotomy with stone removal, ileocecectomy if stones are still present, and later consideration of cholecystectomy plus fistula repair [7]. Low-risk patients can undergo one-stage enterolithotomy, cholecystectomy, and fistula repair, or a two-stage approach in which cholecystectomy and fistula repair follow initial enterolithotomy. For fistula repair itself, options include primary closure, omental patch, jejunoduodenostomy, or pancreatoduodenectomy [7]. This algorithm is somewhat more aggressive in its management in terms of readiness to move to a surgical approach, but more data is needed to determine whether this versus more conservative management will ultimately lead to improved patient outcomes.

## Conclusions

In the end, especially considering the dearth of data regarding outcomes in this rare condition, decisions regarding management must be tailored to the individual patient’s health status, goals, and values. In our patient’s case, her underlying decompensated cirrhosis represented a significant hurdle in terms of surgical risk and ultimately necessitated potential cholecystectomy being deferred, pending further management of her cirrhosis through either transplant or TIPS. Unfortunately, she passed away before definitive management could be achieved. This represents one of the common challenges faced in managing Bouveret syndrome, significant patient comorbidity, which makes the creation of definitive guidelines difficult and tends to push management toward an individually tailored approach.
